# Supplementary material for: Identification of photoperiod-induced specific miRNAs in the adrenal glands of Sunite sheep (Ovis aries)
Source: Front Vet Sci. 2022 Jul 22;9:888207. doi: 10.3389/fvets.2022.888207 (PMC9354845; doi:10.3389/fvets.2022.888207)
Supplement: Supplementary file 1 [file Data_Sheet_1.ZIP › Supplementary file/miRDeep2 file.zip!/miRDeep2 file/Novel_Structure/LP42A2/Novel_422.pdf]

Provisional ID : NC_019460.2_8148
Score total : 2.6
Score for star read(s) : -1.3
Score for read counts : 0
Score for mfe : 2.3
Score for randfold : 1.6
Score for cons. seed :
Total read count : 87
Mature read count : 87
Loop read count : 0
Star read count : 0

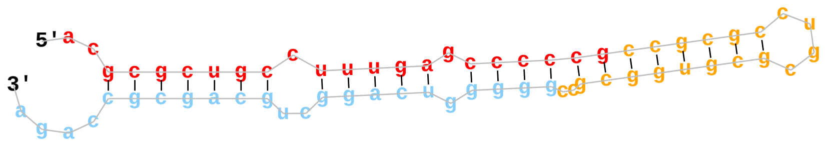

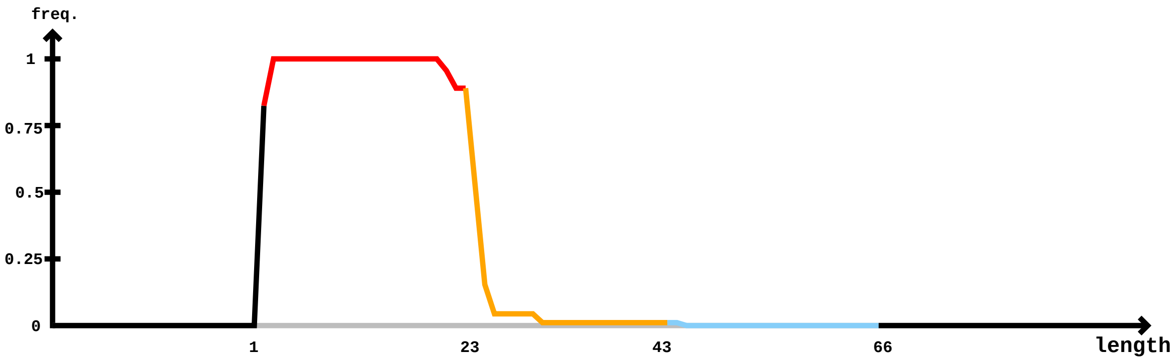

Mature Star

```
5'- ccaaucuaaaugucugaugaacgcgcugccuuugagcccccgccgcgccugcgcguggcgccgggggucaggcugcagcgccagaggacaugcucgcgcauaacuggggucg  -3'   exp
    .........((((((..((...(((((((.(((((.((((((((((((....))))))))..)))).))))).)))))))))..))))))((((...........))))..    reads   mm   sample
    ....................acgcgcugccuuugagccc.........................................................................    4       0    seq
    ....................acgcgcugccuuugagcccc........................................................................    3       0    seq
    ....................acgcgcugccuuugagcccccg......................................................................    35      0    seq
    ....................acgcgcugccuuugagcccccgc.....................................................................    21      0    seq
    ....................acgcgcugccuuugagcccccgcc....................................................................    9       0    seq
    ....................acgcgcugccuuugagcccccgccgcgcc...............................................................    2       0    seq
    ....................acgcgcugccuuugagcccccgccgcgccugcgcguggcgccgg................................................    1       0    seq
    .....................cgcgcugccuuugagcccc........................................................................    3       0    seq
    .....................cgcgcugccuuugagcccccg......................................................................    7       0    seq
    .....................cgcgcugccuuugagcccccgc.....................................................................    4       0    seq
    .....................cgcgcugccuuugagcccccgcc....................................................................    1       0    seq
    .....................cgcgcugccuuugagcccccgccgcgcc...............................................................    1       0    seq
```
